# Transcriptome analyses provide insights into the expression pattern and sequence similarity of several taxol biosynthesis-related genes in three *Taxus* species

**DOI:** 10.1186/s12870-019-1645-x

**Published:** 2019-01-21

**Authors:** Ting Zhou, Xiujun Luo, Chunna Yu, Chengchao Zhang, Lei Zhang, Yao-bin Song, Ming Dong, Chenjia Shen

**Affiliations:** 1 College of Life and Environmental Sciences, Hangzhou Normal University, Hangzhou, 310036 China; 2 Zhejiang Provincial Key Laboratory for Genetic Improvement and Quality Control of Medicinal Plants, Hangzhou Normal University, Hangzhou, 310036 China; 3 Department of Plant Pathology, Washington State University, Pullman, WA 99164-6430 USA; 4 Key Laboratory of Hangzhou City for Ecosystem Protection and Restoration, College of Life and Environmental Sciences, Hangzhou Normal University, Hangzhou, 310036 China

**Keywords:** Expression pattern, Taxol, Taxoid, *Taxus*, Transcriptome

## Abstract

**Background:**

Taxol is an efficient anticancer drug; however, the accumulation of taxoids can vary hugely among *Taxus* species. The mechanism underlying differential accumulation of taxoids is largely unknown. Thus, comparative analysis of the transcriptomes in three *Taxus* species, including *T. media*, *T. mairei* and *T. cuspidata*, was performed.

**Results:**

KEGG enrichment analysis revealed that the diterpenoid biosynthesis and cytochrome P450 pathways were significantly enriched in different comparisons. Differential expressions of these taxol biosynthesis related genes might be a potential explanation for the interspecific differential accumulation of taxol and its derivatives. Besides, the sequences of several MEP pathway-associated genes, such as *DXS*, *DXR*, *MCT*, *CMK*, *MDS*, *HDS*, *HDR*, *IPPI*, and *GGPPS*, were re-assembled based on independent transcriptomes from the three *Taxus* species. Phylogenetic analysis of these MEP pathway-associated enzymes also showed a high sequence similarity between *T. media* and *T. cuspidata*. Moreover, 48 JA-related transcription factor (TF) genes, including 10 *MYB*s, 5 *ERF*s, 4 *RAP*s, 3 *VTC*s, and 26 other TFs, were analyzed. Differential expression of these JA-related TF genes suggested distinct responses to exogenous JA applications in the three *Taxus* species.

**Conclusions:**

Our results provide insights into the expression pattern and sequence similarity of several taxol biosynthesis-related genes in three *Taxus* species. The data give us an opportunity to reveal the mechanism underlying the variations in the taxoid contents and to select the highest-yielding *Taxus* species.

**Electronic supplementary material:**

The online version of this article (10.1186/s12870-019-1645-x) contains supplementary material, which is available to authorized users.

## Background

Taxol (generic name paclitaxel), very important for certain types of cancer treatments, was first isolated from the bark of the Pacific yew *Taxus brevifolia* and gained marketing approval from the U.S. Food and Drug Administration for the treatment of various cancers [1]. Limited by several barriers, such as the slow growth of wild yews, destructive harvesting techniques, and complicated purification procedures, the demand for taxol exceeds the supply [2].

The metabolic pathway of taxol biosynthesis has been partially revealed. Firstly, three units of the C5 isoprenoid precursor isopentenyl diphosphate (IPP) and one unit of dimethylallyl diphosphate, which are produced by the plastidial 2-C-methyl-D-erythritol phosphate (MEP) pathway, are used to synthesize geranylgeranyl diphosphate (GGPP), the precursor of the diterpenoid taxane core [3]. The cyclization of GGPP to taxa-4(5),11(12)-diene is conducted by taxadiene synthase, which catalyzes a slow, but not rate-limiting step [4, 5]. Then, the pathway toward taxol involves many intermediate enzymatic steps, including eight hydroxylations, five acyl/aroyl transfer reactions, one epoxidation, one oxidation, two CoA esterifications and one *N*-benzoylation [6]. For example, several cytochrome P450s, including 2α-, 5α-, 7β-, 9α-, 10β-, 13α- and 14β-hydroxylases, participate in the oxygenation steps of taxadiene [7]. The assembly of the C13-side chain appended to baccatin III is considered to be the final step of the taxol biosynthesis pathway [8].

RNA-seq has been frequently used to evaluate the expression differences among different *Taxus* species. The first transcriptome of the *Taxus* genus was obtained from the cultured cells of *T. cuspidata* in 2010, and then the organ-specific transcriptomes of *T. mairei* were published in the same and next year [9, 10]. The deep sequencing of different *Taxus* species indicated roles of methyl-jasmonate (MeJA) in the regulation of the terpenoid biosynthesis pathway, which supplies the precursors for taxol biosynthesis [11–13]. A comparison between *T. media* and *T. mairei* transcriptomes provided insights into the differential expressions of candidate genes involved in the taxoid biosynthetic pathways [14]. Transcriptome profiles of *T. chinensis* under different temperatures revealed an involvement of various TF families, such as NAC, WRKY, bZIP, MYB, and ERF families, in the responses of Chinese yews to cold stress [15]. Recently, transcriptome assembly and systematic identification of the cytochrome P450 and WRKY families in *T. chinensis* have been performed by Yu’s group [16, 17]. In a novel *T. yunnanensis* cultivar, transcriptome profiling illustrated a specific regulation mechanism of taxol biosynthesis [18].

Recent technical advances in the large-scale identification of genes have revealed several complex processes involved in the regulation of plant metabolism [19, 20]. In nature, the amount of each taxoid varied among varieties and species [21–23]. Thus, investigations into the variations in the expression pattern and sequence similarity of the key genes involved in the metabolism of taxoids in three different *Taxus* species will provide an opportunity to select the highest-yielding species and to elucidate the mechanism underlying the species-related variations in the taxoid contents.

## Methods

### Plant materials and RNA extraction

**Fig. 1:**
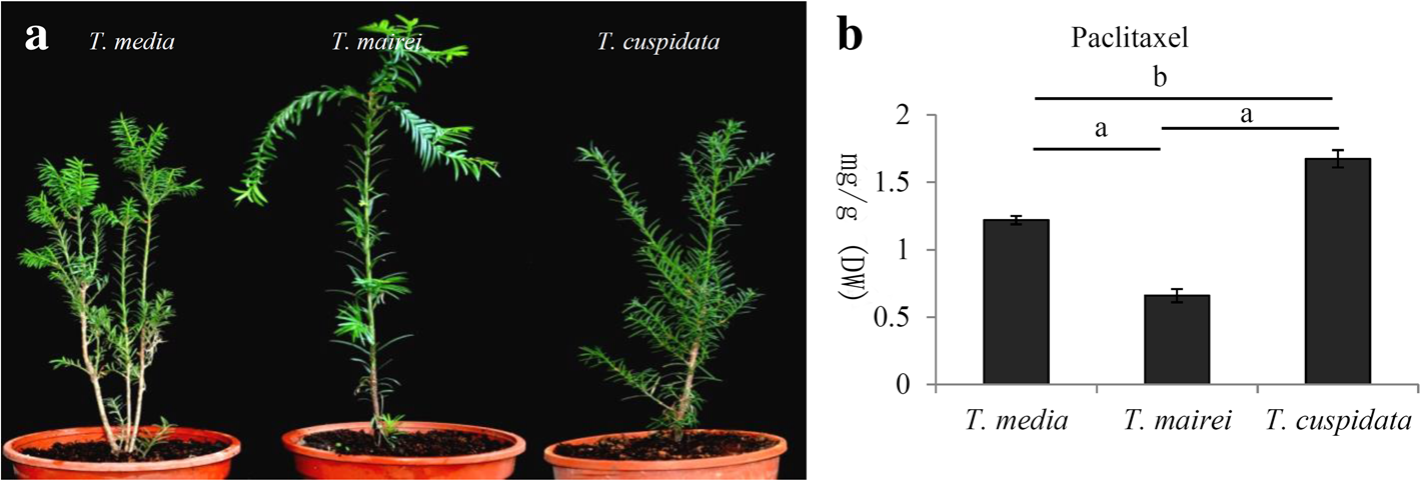
Variation of the contents of taxoids among three different *Taxus* species. **a** A picture of *T. media*, *T. mairei* and *T. cuspidata* under greenhouse condition. Fresh twigs were harvested from three cultivated *Taxus* species. **b** The contents of paclitaxel were quantified by HPLC-MS/MS method. A *P* value < 0.05 was considered to be statistically significant and indicated by “b” and *P* < 0.01 was indicated by “a”

Fresh twig samples were harvested from three-year-old cultivated *Taxus* trees, including *T. media*, *T. mairei*, and *T. cuspidata*, in March 2015 in a growth chamber of Hangzhou Normal University, Hangzhou, China (Fig. 1a). The growth conditions were set at 25 ± 1 °C with a light/dark cycle of 12/12 h and a 55–65% relative humidity. Five independent trees for each species were used in our study.

For RNA isolation, we used the methodology previously described by Yu et al. [14]. In detail, total RNAs were extracted using an RNeasy plant mini kit (Qiagen, Hilden, Germany) according to its manual. DNA contamination was cleaned by adding DNase I to the mixture. The clean RNAs were checked using 1% agarose gel electrophoresis. The quality of total RNAs was confirmed using an RNA 6000 Nano LabChip kit (Agilent, Santa Clara, CA, USA) with an RNA integrity number > 7.0.

### Library preparation and transcriptomic analysis

Total RNA samples of 10 μg from each RNA extract (3 species × 3 biological replicates) were prepared. The methods of library preparation, de novo strategy and transcriptomic analysis were the same to our previous published work [14]. In brief, RNA representing a specific mRNA was subjected to oligo (dT) attached magnetic beads. Then, purified RNAs were fragmented into small pieces. These small fragments were reverse-transcribed to create cDNA libraries using a sample preparation kit (Illumina, San Diego, USA). Sequencing was carried out using an Illumina Hiseq 4000 platform (LC-Bio, Hangzhou, China) according to its protocol.

Three important parameters, including Q20, Q30 and GC content, were verified to evaluate all the reads using FastQC online tool (http://www.bioinformatics.babraham.ac.uk/projects/fastqc/). For de novo assembly, the transcriptome from each species was processed using software Trinity 2.4.0 [24]. For gene identification and expression analysis, the reads from different species were co-assembled, and for gene sequence analysis, the reads from different species were assembled separately. The raw sequence data has been submitted to the NCBI Short Read Archive with accession number GSE121523.

### Functional annotation and enrichment analysis

For gene annotation, we used the methodology previously described by Yu et al. [14]. In detail, all assembled unigenes were searched against various databases, including the non-redundant (Nr) protein, Gene Ontology (GO), SwissProt, Kyoto Encyclopedia of Genes and Genomes (KEGG), and eggNOG databases, with a threshold of *E* value < 0.00001. GO and KEGG enrichment analysis were performed on the DEGs by perl scripts in house.

### Differentially expressed unigene (DEGs) analysis

Expression levels for each unigene were calculated using the TPM method [25]. The DEGs were screened with criterions: ǀlog2(fold change) ǀ > 1 and statistical significance *P* < 0.05. The transcript abundance values were transformed into Z-score after log transformation. The heatmap was drawn using MultiExperiment Viewer (version 4.9.0) basing on their log_2_ values of transformed Z-scores.

### Homology analysis and phylogenetic tree building

Gene homology analysis was carried out using ClustalW with default parameters. Predicted full-length protein sequences of the key genes involved in the taxol biosynthesis pathway were used for alignments. The results were subsequently visualized by the GeneDoc software and a tree was constructed using software MEGA6.1 employing NJ method.

### Analysis of paclitaxel contents

Paclitaxel (≥ 99%; CAS No. 33069–62-4) was purchased from Aladdin Biochemical Technology (Shanghai, China). Fresh twigs of each sample were collected, dried at 40 °C for 3 d, and powdered. A previously published method was used to prepare crude extracts and paclitaxel determination [14]. The quantification of paclitaxel was presented as the means of at least three replicates ± standard error.

### Statistical analysis

For the transcriptome analyses, the false discovery rate was used to calculate the resulting *P* values, and the *P* values were adjusted using the Benjamini and Hochberg method. Statistical analyses were performed using SPSS software version 19.0 (SPSS Inc., Chicago, IL, USA), and an ANOVA was applied to compare the differences between two groups.

## Results

### Variations in taxol contents using a HPLC-MS/MS approach

To determine more precisely the differences in taxol contents among the three *Taxus* species, a HPLC-MS/MS approach was used. The data showed that *T. cuspidata* contained the highest level of taxol (1.67 mg.g^− 1^, DW) and *T. mairei* contained the lowest levels of taxol (0.66 mg.g^− 1^, DW) (Fig. 1b).

### Transcriptomes of different *Taxus* species

**Fig. 2:**
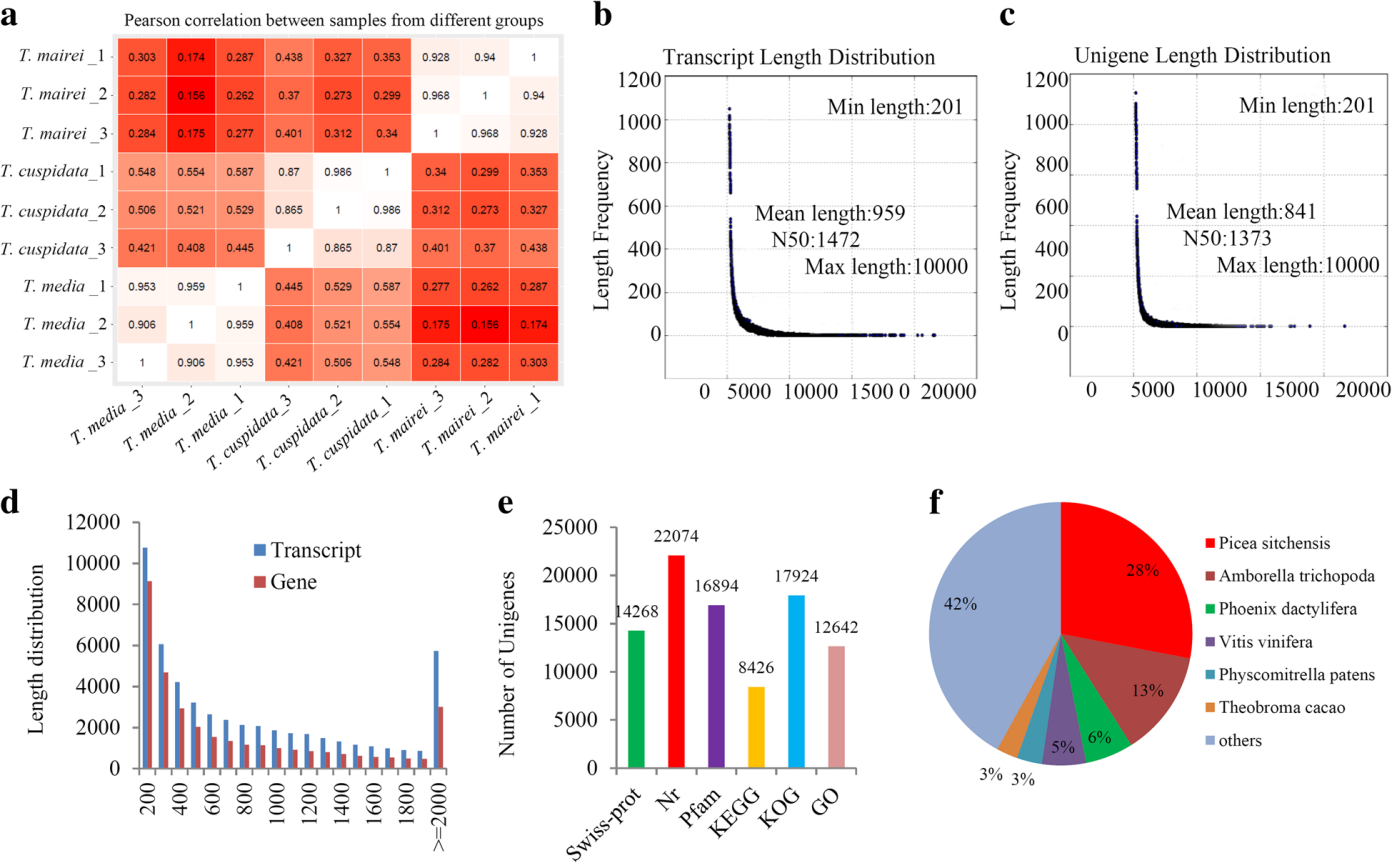
Illumina sequencing and transcriptomes of different *Taxus* species. **a** Pair-wise Pearson’s correlation coefficients of the sequencing data from three replicates × three *Taxus* species. The detail information of the assembled transcripts (**b**) and unigenes (**c**). **d** The size distributions of transcripts and unigenes of *Taxus* species. **e** The annotation of unigenes basing on various databases. **f** The species distribution of the annotated unigenes

The raw reads were qualified, and adapters were removed, yielding 67.49 Gb of sequence data, including 19.84 Gb from *T. media*, 21.90 Gb from *T. cuspidata*, and 25.75 Gb from *T. mairei* (Additional file 1). Pair-wise Pearson’s correlation coefficients of three replicates × three *Taxus* species indicated high repeatability of the sequencing data (Fig. 2a). To obtain an overview of the transcriptomic variations, a principal components analysis (PCA) was performed, and the explained values of PC1 and PC2 were 18.4 and 36.2%, respectively (Additional file 2). The PCA clearly separated the three species into two groups, suggesting a close similarity between *T. media* and *T. cuspidata*.

All of the reads obtained from the three *Taxus* species were assembled, resulting in 52,261 transcripts (N50: 1472), with a mean length of 959 bp (Fig. 2b), and 33,895 unigenes (N50: 1373), with a mean length of 841 bp (Fig. 2c). The size distributions of transcripts and unigenes are shown in Fig. 2d. For transcripts, 10.98% of the reads were > 2000 bp in length, and the majority of the reads (64.06%) were < 1000 bp in length. Only 8.87% of the unigenes were > 2000 bp in length, and the majority of unigenes were between 200 bp and 500 bp in length. In total, 14,268 unigenes were annotated by the Swiss-Prot database, 22,074 unigenes were identified in the Nr database, 16,894 unigenes displayed significant similarities to known proteins in the Pfam database, and 8426 and 17,924 unigenes were annotated in the KEGG and KOG databases, respectively (Fig. 2e). The species distribution of the annotated unigenes is shown in Fig. 2f.

### Identification of the DEGs among different *Taxus* species

**Fig. 3:**
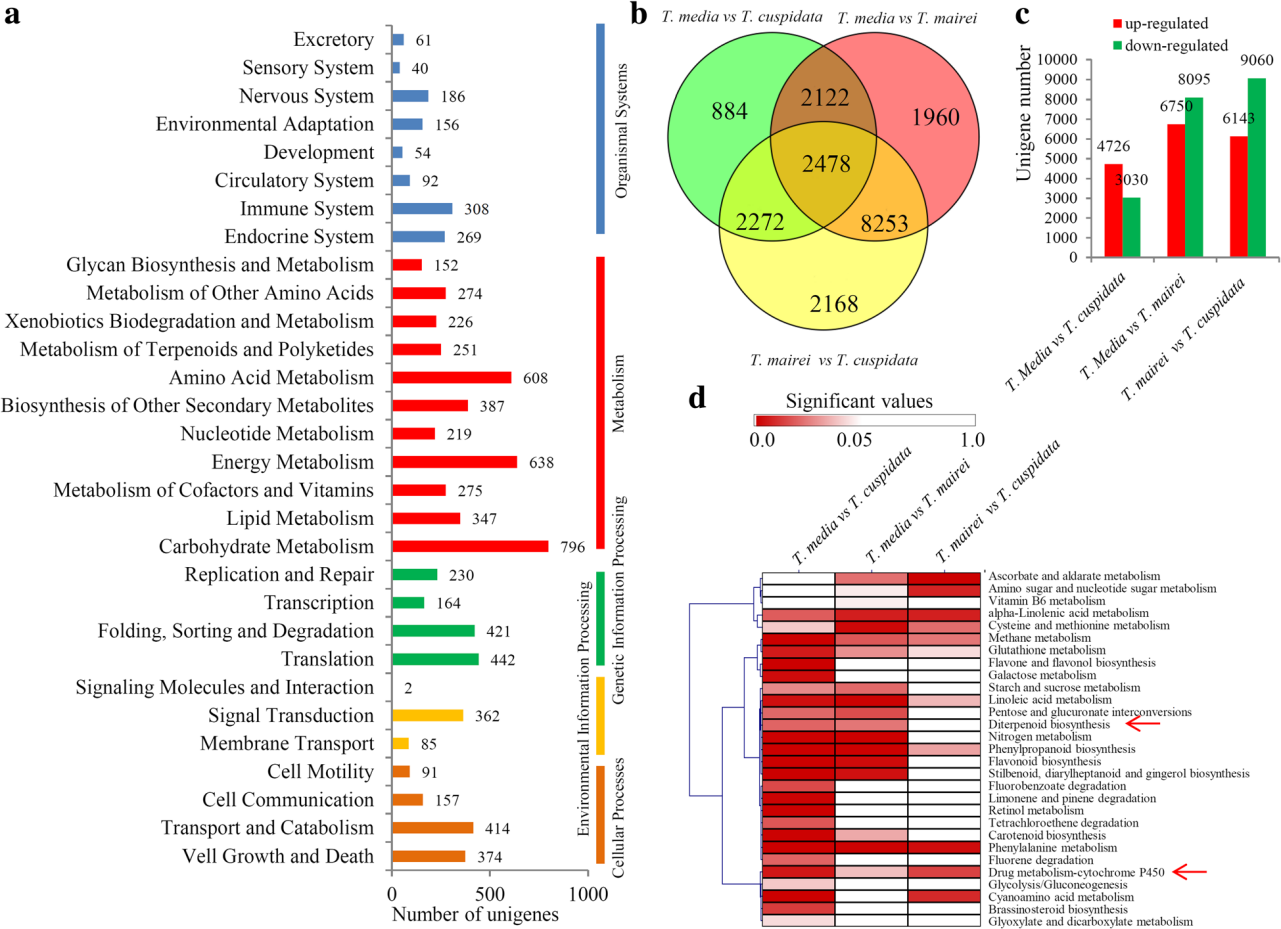
Identification of the DEGs among three different *Taxus* species. **a** Classification of enriched KEGG terms. A large number of unigenes could be classified into various KEGG metabolic and signaling pathways, including pathways related to organismal systems, metabolism, genetic information processing, environmental information processing, and cellular process. **b** A venn diagram showed the numbers of the DEGs in three comparisons, including *T. media* vs *T. cuspidata*, *T. media* vs *T. mairei* and *T. mairei* and *T. cuspidata* comparisons. **c** The numbers of the up- and down-regulated unigenes in the three comparisons. **d** KEGG enrichment analysis of the DEGs in the three comparisons. The significant *P* values of each KEGG term in the three comparisons were shown by a heatmap. The bar indicated the significant values. Red arrows indicated two metabolic pathways involved in the taxol biosynthesis pathway

In our study, a large number of unigenes were classified into various KEGG metabolic and signaling pathways (Additional file 3). The most enriched KEGG pathways were ‘amino acid metabolism’ (608 unigenes), ‘energy metabolism’ (638 unigenes), and ‘carbohydrate metabolism’ (796 unigenes) (Fig. 3a).

The numbers of DEGs identified in each comparison are shown in a Venn diagram (Fig. 3b). In detail, 4726 *T. media* highly-expressed unigenes and 3030 *T. cuspidata* highly-expressed unigenes were identified in the *T. media* vs. *T. cuspidata* comparison. In the *T. media* vs. *T. mairei* comparison, 6750 unigenes highly expressed in *T. media* and 8095 unigenes highly expressed in *T. mairei*. In the *T. mairei* vs. *T. cuspidata* comparison, 6143 *T. mairei* highly-expressed unigenes and 9060 *T. cuspidata* highly-expressed unigenes were identified (Fig. 3c).

Among these pathways, 26 pathways were significantly enriched (*P* < 0.05) in the *T. media* vs. *T. cuspidata* comparison. In the *T. media* vs. *T. mairei* comparison, 18 pathways were significantly enriched, while in the *T. mairei* vs. *T. cuspidata* comparison, only 11 pathways were significantly enriched (Fig. 3d).

### DEGs associated with the taxol biosynthesis pathway

**Fig. 4:**
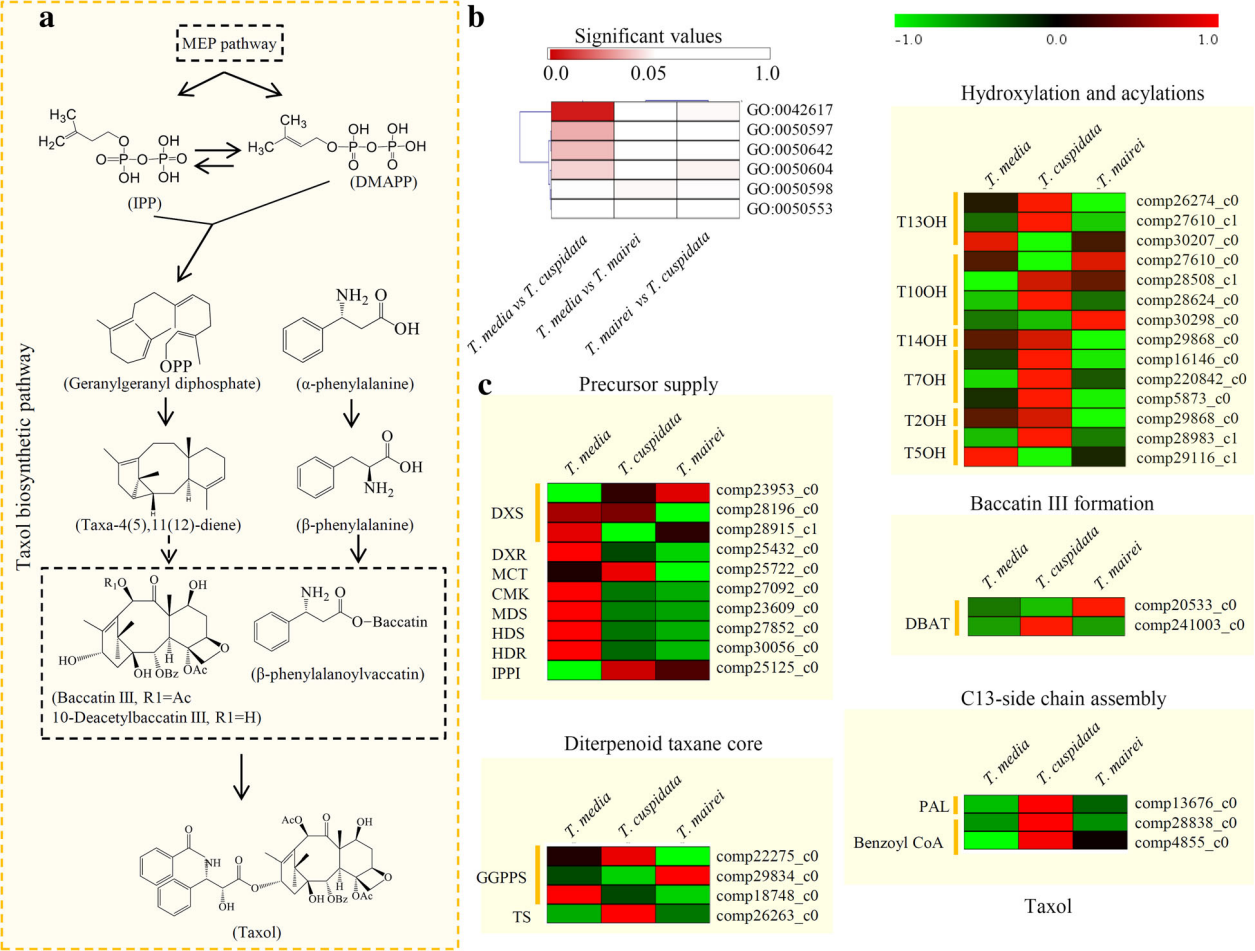
Differential expression of the unigenes related to the taxol biosynthesis pathway. **a** Overview of the taxol biosynthesis pathway. **b** Enrichment analysis of six taxol biosynthesis-related GO terms. The significant *P* values of each GO term in the three comparisons were shown by a heatmap. **c** Expression analysis of the unigenes related to the taxol biosynthesis pathway. Enzymes abbreviations are: DXS: 1-Deoxy-D-xylulose 5-phosphate synthase; DXR: 1-Deoxy-D-xylulose 5-phosphate reductoisomerase; MCT: 2-C-methyl-D-erythritol 4-phosphate cytidylyltransferase; CMK: 4-(Cytidine 5-diphospho)-2-C-methyl-D-erythritol kinase; MDS: 2-C-methyl-D-erythritol 2,4-cyclodiphosphate synthase; HDS: 4-Hydroxy-3-methylbut-2-enyl-diphosphate synthase; HDR: 4-Hydroxy-3-methylbut-2-enyl diphosphate reductase; IPPI: isopentenyl diphosphate isomerase; GGPPS: geranylgeranyl diphosphate synthase; TS, taxadiene synthase; T13OH: taxoid 13-α-hydroxylase; T10OH: taxoid 10-β-hydroxylase; T14OH: Taxoid 14-β-hydroxylase; T7OH: taxoid-7β-hydroxylase; T2OH: taxoid-2α-hydroxylase; T5OH: taxadiene-5α-hydroxylase; DBAT: 10-deacetylbaccatin III-10-β-O-acetyltransferase; PAL: phenylalanine aminomutase; Benzoyl CoA: 2-debenzoyl-7,13-diacetylbaccatin III-2-O-benzoyl transferase. The bar indicated the “log2(expression folds)”

For taxol biosynthesis, several intermediate steps, including precursor supply, diterpenoid taxane core synthesis, hydroxylations, acylations, baccatin III formation, and C13-side chain assembly, were involved (Fig. 4a) [6]. A transcriptomic analysis revealed six taxol biosynthesis-related GO terms, including ‘paclitaxel biosynthetic process’ (GO:0042617), ‘taxane 10-beta-hydroxylase activity’ (GO:0050597), ‘2-alpha-hydroxytaxane 2-O-benzoyltransferase activity’ (GO:0050642), ‘taxadiene 5-alpha-hydroxylase activity’ (GO:0050604), ‘taxane 13-alpha-hydroxylase activity’ (GO:0050598), and ‘taxadiene synthase activity’ (GO:0050553), and provided an opportunity to analyze the differential expression of taxol biosynthesis pathway-related genes among different *Taxus* species. In detail, the genes in four GO terms, including GO:0042617 (*P* = 1.05E-9), GO:0050597 (*P* = 3.21E-4), GO:0050642 (*P* = 1.11E-3) and GO:0050604 (*P* = 8.36E-3), significantly changed in the *T. media* vs. *T. cuspidata* comparison (Fig. 4b).

Furthermore, the expression patterns of the taxol biosynthesis pathway-related genes were investigated. In the MEP pathway, most of the genes showed highest expression levels in *T. media*. For the diterpenoid taxane core biosynthesis, the gene (comp26263_c0) encoding the key enzyme, taxadiene synthase, predominantly expressed in *T. cuspidata*. For the hydroxylation and acylations steps, a series of taxane hydroxylase-encoding genes was identified, and most of them highly expressed in *T. cuspidata*. For the C13-side chain assembly, one phenylalanine ammonia-lyase encoding gene (com13676_c0) and two benzoyl-CoA encoding genes (comp28838_c0 and comp4855_c0) were identified, and they predominantly expressed in *T. cuspidata* (Fig. 4c).

### Phylogenetic analysis of the MEP pathway-associated proteins

From the independently assembled transcriptomes of three *Taxus* species, the full-length sequences of 12 MEP pathway-related genes were obtained. Three predicted peptide sequences of each key enzyme were used for phylogenetic tree construction. For most of the MEP pathway-related enzymes, including 1-deoxy-D-xylulose 5-phosphate reductoisomerase (DXR), 1-deoxy-D-xylulose 5-phosphate synthase 1 (DXS1), DXS2, 2-C-methyl-D-erythritol 2,4-cyclodiphosphate synthase (MDS), 4-(cytidine 5-diphospho)-2-C-methyl-D-erythritol kinase (CMK), GPPS, geranylgeranyl diphosphate synthase 1 (GGPPS1), and GGPPS2, sequences from *T. cuspidata* were highly similar to those from *T. media*. The sequences of three MEP pathway-related enzymes, 4-hydroxy-3-methylbut-2-enyl diphosphate reductase (HDR), 4-hydroxy-3-methylbut-2-enyl-diphosphate synthase (HDS), and isopentenyl diphosphate isomerase (IPPI), displayed high similarity levels between *T. media* and *T. cuspidata* (Additional file 4).

### Transcriptomic analysis reveals the differences in jasmonic acid (JA) pathway-related genes

**Fig. 5:**
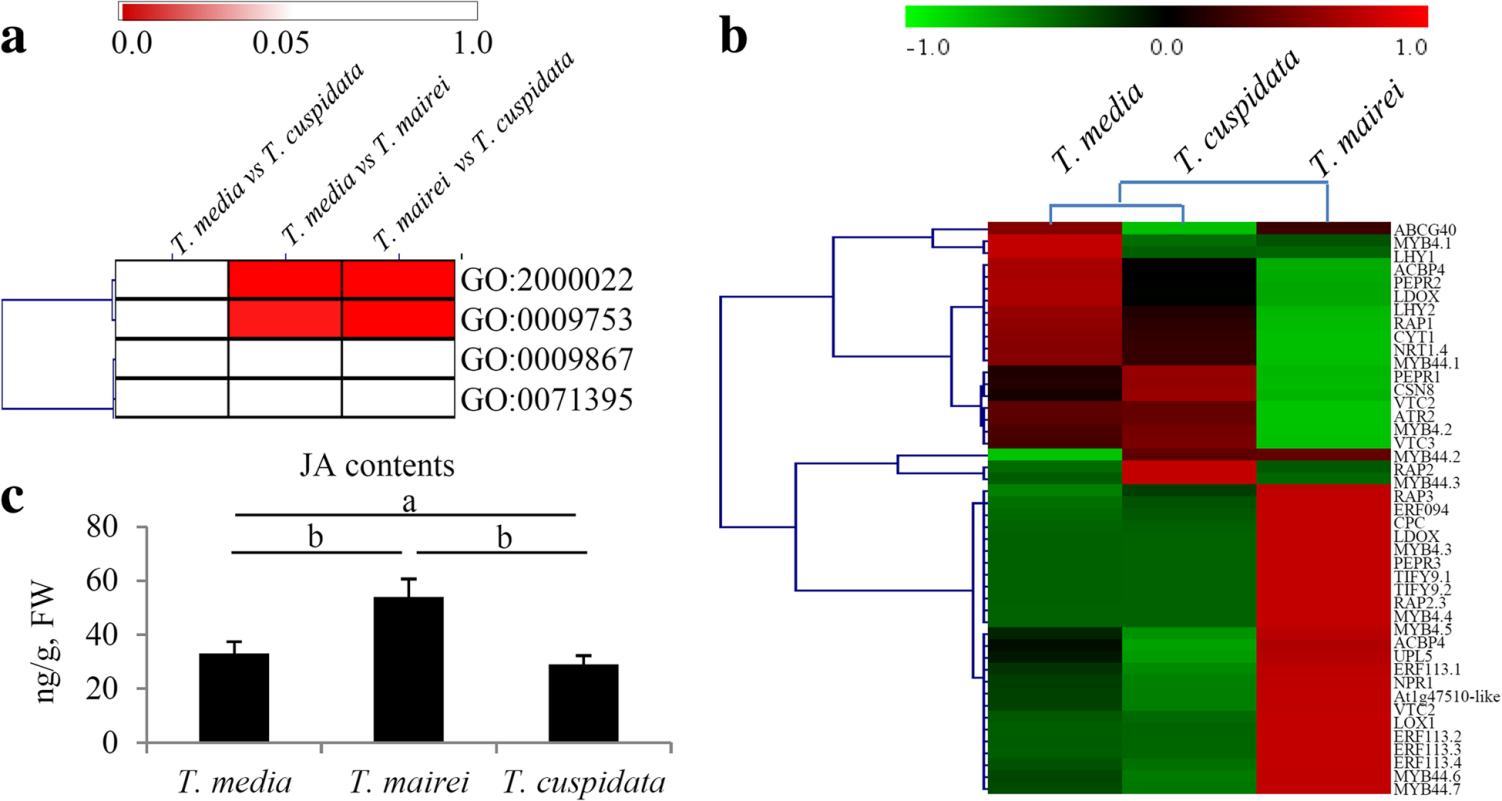
Analysis of transcriptome reveals the differences in JA pathway-related genes. **a** Significance analysis of five JA-related GO terms in three comparisons, including *T. media* vs *T. cuspidata*, *T. media* vs *T. mairei* and *T. mairei* and *T. cuspidate* comparisons, were shown by a heatmap. **b** Expression profiles of the transcription factor encoding genes related to JA signaling pathway were showed by a heatmap. **c** Determination of endogenous JA contents in three *Taxus* species. A *P* value > 0.05 was considered to be statistically significant and indicated by “b” and *P* < 0.05 was indicated by “a”

Based on the transcriptomes, four JA metabolism-related GO terms, ‘regulation of jasmonic acid mediated signaling pathway’ (GO:2000022), ‘response to jasmonic acid stimulus’ (GO:0009753), ‘jasmonic acid mediated signaling pathway’ (GO:0009867), and ‘cellular response to jasmonic acid stimulus’ (GO:0071395), were identified. In detail, the significant values of GO:2000022 in the *T. mairei* vs. *T. cuspidata* and *T. media* vs. *T. mairei* comparisons were 0.044 and 0.034, respectively. For GO:0009753, the significant values in the *T. mairei* vs. *T. cuspidata* and *T. media* vs. *T. mairei* comparisons were 0.048 and 0.033, respectively (Additional file 5). There were no significant differences in these terms in the *T. media* vs. *T. cuspidata* comparison. Significant differences for GO:2000022 and GO:0009753 were observed in the *T. media* vs. *T. mairei* and *T. mairei* vs. *T. cuspidata* comparisons (Fig. 5a). The expression pattern of 48 JA metabolism- and signaling pathway-related genes was analyzed (Fig. 5b). Interestingly, the expression pattern of these JA-related genes in *T. media* was similar to that in *T. cuspidata* (Additional file 6). Furthermore, the endogenous JA contents were determined in the three *Taxus* species. Significant differences in the endogenous JA content were observed in *T. media* vs. *T. mairei* and *T. mairei* vs. *T. cuspidata* comparisons (Fig. 5c).

### Identifications of TFs families in *Taxus* species

A number of TFs were reported to play important roles in taxol biosynthesis. In our study, 736 putative TF encoding genes belonging to 17 major TF families were analyzed in *Taxus* (Additional file 7). A large number of TFs were included in the MYB family (174 genes), ARF family (90 genes), and WRKY family (61 genes). The numbers of differential expressed TFs in the three comparisons were showed in Additional file 8. To screen key regulators for taxol biosynthesis, the expression levels of all TF genes were showed in a heatmap (Additional file 9).

## Discussion

Because *Taxus* plants are the major natural resource for taxol, comprehensive phytochemical analyses of *Taxus* species have been performed [6, 26]. A large number of compounds have been identified in various *Taxus* species [27]. However, the levels of taxoids accumulation may vary significantly among species. The previous study showed that the taxol contents in *T. mairei*, *T. media*, and *T. cuspidata* needles were 0.163 mg.g^− 1^, 0.435 mg.g^− 1^, and 0.249 mg.g^− 1^, respectively, by UFLC-ESI-MS and UFLC-DAD analysis [28]. In our study, *T. mairei* contained the lowest levels of taxol, which was in agree with the results from the previous work. However, the contents of taxol in *T. cuspidata* was higher than that in *T. media*. Another previous study showed that the taxol contents ranged from 0.1 to 0.4 mg.g^− 1^ in *T. mairei* needles in the course of a year [29]. Rikhari’s group showed that taxol content in the bark of Himalayan yew was related to tree age and sex [30]. The accumulation of taxol was greatly affected by natural environment and cultivation conditions, thus the taxol contents varied among different studies.

A substantial number of DEGs, among which 26 major metabolic pathways were enriched, were identified in the *T. media* vs. *T. cuspidata*, *T. media* vs. *T. mairei*, and *T. mairei* vs. *T. cuspidata* comparisons (Fig. 3b, c). Formation of diterpenoid taxane skeleton is an essential step for taxol biosynthesis, and it occurs before the cyclization of taxane skeleton [4]. In our study, the diterpenoid biosynthesis pathway significantly changed in the *T. media* vs. *T. cuspidata* and *T. media* vs. *T. mairei* comparisons. No significant changes were observed in the *T. mairei* vs. *T. cuspidata* comparison (Fig. 3d). It suggested that the genes involved in diterpenoid biosynthesis shared a similar expression pattern between *T. mairei* and *T. cuspidata*. Additionally, the drug metabolism–cytochrome P450 pathway, containing a series of taxoid oxygenases, was significantly different in all three comparisons [7, 31]. The differential expressions of the above two important metabolic pathways might provide a number of genes that were involved in the interspecific differential accumulations of taxol and its derivatives. In the taxol biosynthetic pathway, α- and β-phenylalanine are involved in the side chain assembly [32, 33]. KEGG analysis showed that the phenylalanine metabolism pathway was siginificantly enriched in all three comparisons, suggesting an involvement of side chain assembly in interspecific differential accumulations of taxol. Additionally, flavonoids and phenylpropanoids are important active ingredients isolated from *Taxus* reminder extracts [23, 34, 35]. Two enriched flavonoid metabolism-related pathways, including the flavonoid biosynthesis and flavone and flavonol biosynthesis pathways, were identified in the *T. media* vs *T. cuspidata* comparison. Our data indicated that there were great differences in the accumulation of flavonoids between *T. media* and *T. cuspidata*. The phenylpropanoid biosynthesis pathway was enriched in all three comparisons, suggesting deep variations in various active ingredient metabolisms, not only taxol, among different *Taxus* species.

Previous studies identified a number of taxol biosynthesis-related genes that were assigned into six GO terms [12, 36]. In the *T. baccata* plantlets, the taxane contents were correlated with the expression levels of *TXS*, *DBAT*, *BAPT*, and *DBTNBT* genes [37]. In our study, there was a great difference in the expression of taxol biosynthesis-related genes between *T. media* and *T. cuspidata* (Fig. 4b). The expression pattern of the taxol biosynthesis-related genes, including precursor supply (10 genes), diterpenoid taxane core (4 genes), hydroxylation and acylations (14 genes), baccatin III formation (2 genes), and C13-side chain assembly (3 genes), was investigated. The dynamic expression levels of these genes might provide a potential explanation for the interspecific differential accumulation of taxol.

The sequences and structural properties of key enzymes in the taxol biosynthesis pathway, which result in different catalytic efficiencies, are closely related to taxol production [38, 39]. For example, improvement of the 10-deacetylbaccatin III-10-β-O-acetyltransferase (DBAT) catalytic fitness contributes to the abundant accumulation of baccatin III [40]. Sequences of several key enzymes were re-assembled based on the independent transcriptomes from each *Taxus* species. Due to the unavailability of corresponding genome data, the assembled sequences of most taxol pathway-related genes were imperfect. Fortunately, the full-length sequences of most MEP pathway genes were available. Interestingly, for most MEP pathway genes, such as *DXR*, *DXS*, *MDS*, *CMK*, *GPPS*, and *GGPPS*, greater sequence similarities were observed between *T. cuspidata* and *T. media*. This result was consistent with the differential accumulation of taxol among the three *Taxus* species.

JA and MeJA have been widely applied to increase secondary metabolite production in various culture systems [41]. Exogenous MeJA has also been used as an effective elicitor to increase the production of paclitaxel and its derivatives in *Taxus* cell-suspension cultures [11, 42]. Considering the highest accumulation of endogenous JA in *T. mairei*, the level of endogenous JAs may not reach the threshold to play a role in taxol biosynthesis. Several TFs were involved in the JA-mediated transcriptional regulation of secondary metabolism in medicinal plants [43]. In our study, a number of JA pathway-related genes were identified, and their expression levels were also analyzed in the three *Taxus* species. Among these genes, some TFs, such as MYB, LHY, and ERF, were included (Fig. 5b). The differential expression of these JA-related TFs suggested distinct responses of the three *Taxus* species to exogenous MeJA application.

Furthermore, increasing evidences showed that various TF families, such as bHLH and WRKY, involved in the regulation of taxol biosynthesis [32]. For example, TcMYC1, TcMYC2, and TcMYC4 are involved in transduction of JA signals and regulation of taxol biosynthesis [44, 45]. A WRKY TF, TcWRKY1, plays a role in transcriptional activation of *DBAT* in *T. chinensis* [46]. In our study, 43 bHLH family and 61 WRKY family TFs were identified. More than half of these bHLH TFs and most of these WRKY TFs were identified as DEGs in the *T. media* vs. *T. cuspidata* and *T. media* vs. *T. mairei* comparisons, providing a number of candidate regulators of taxol biosynthesis.

## Conclusions

Differential expressions of the genes involved in taxol biosynthesis pathway, including precursor supply-, diterpenoid taxane core-, hydroxylation and acylation-, baccatin III formation-, and C13-side chain assembly-related genes, might provide a potential explanation for the interspecific differential accumulation of taxol in different *Taxus* species. Phylogenetic analysis indicated that sequence diversity in the MEP pathway genes may be another factor that determines the variations in taxoids. Our results contribute to a deeper understanding of the interspecific differential accumulation of taxoids in three *Taxus* species.

## Additional files

Additional file 1: **Table S1.** The detail information of raw reads from different sample groups. (XLSX 9 kb)

Additional file 2: **Figure S1.** Principal components analysis of the three transcriptomes. (DOCX 14 kb)

Additional file 3: **Table S2.** KEGG classification of the unigenes. (XLSX 580 kb)

Additional file 4: **Figure S2.** Phylogenetic analysis of the proteins associated with the MEP pathway. (DOCX 412 kb)

Additional file 5: **Table S3.** The significant values of each JA-related GO terms in various comparisons. (XLSX 9 kb)

Additional file 6: **Table S4.** The expression levels of the JA-related genes in different *Taxus* species. (XLSX 17 kb)

Additional file 7: **Table S5.** Identifications of transcription factors of the three *Taxus* species. (XLSX 205 kb)

Additional file 8: **Table S6.** The numbers of differential expressed TFs in the three comparisons. (XLSX 9 kb)

Additional file 9: **Figure S3.** A heatmap of differential expressed TF genes in the three comparisons. (DOCX 283 kb)

## Abbreviations

10-DAB: 10-deacetylbaccatin-III
ANOVA: Analysis of variance
CMK: 4-(Cytidine 5-diphospho)-2-C-methyl-D-erythritol kinase
DEG: Differential expressed genes
DXR: 1-Deoxy-D-xylulose 5-phosphate reductoisomerase
DXS1: 1-Deoxy-D-xylulose 5-phosphate synthase 1
GGPP: Geranylgeranyl diphosphate
GO: Gene Ontology
JA: Jasmonic acid;
KEGG: Kyoto Encyclopedia of Genes and Genomes
MDS: 2-C-methyl-D-erythritol 2,4-cyclodiphosphate synthase
MeJA: Methyl-jasmonate
MEP: 2-C-methyl-D-erythritol phosphate
UPLC-MS/MS: Ultra Performance Liquid Chromatography-Coupled Mass Spectrometry
