# Supplementary material for: Transcriptome analyses provide insights into the expression pattern and sequence similarity of several taxol biosynthesis-related genes in three Taxus species
Source: BMC Plant Biol. 2019 Jan 21;19:33. doi: 10.1186/s12870-019-1645-x (PMC6341696; doi:10.1186/s12870-019-1645-x)
Supplement: Supplementary file 2 — Figure S1. Principal components analysis of the three transcriptomes. (DOCX 14 kb) [file 12870_2019_1645_MOESM2_ESM.docx]

Figure S1 Principal components analysis of the three transcriptomes.
