# Supplementary material for: Transcriptome analyses provide insights into the expression pattern and sequence similarity of several taxol biosynthesis-related genes in three Taxus species
Source: BMC Plant Biol. 2019 Jan 21;19:33. doi: 10.1186/s12870-019-1645-x (PMC6341696; doi:10.1186/s12870-019-1645-x)
Supplement: Supplementary file 4 — Figure S2. Phylogenetic analysis of the proteins associated with the MEP pathway. (DOCX 412 kb) [file 12870_2019_1645_MOESM4_ESM.docx]

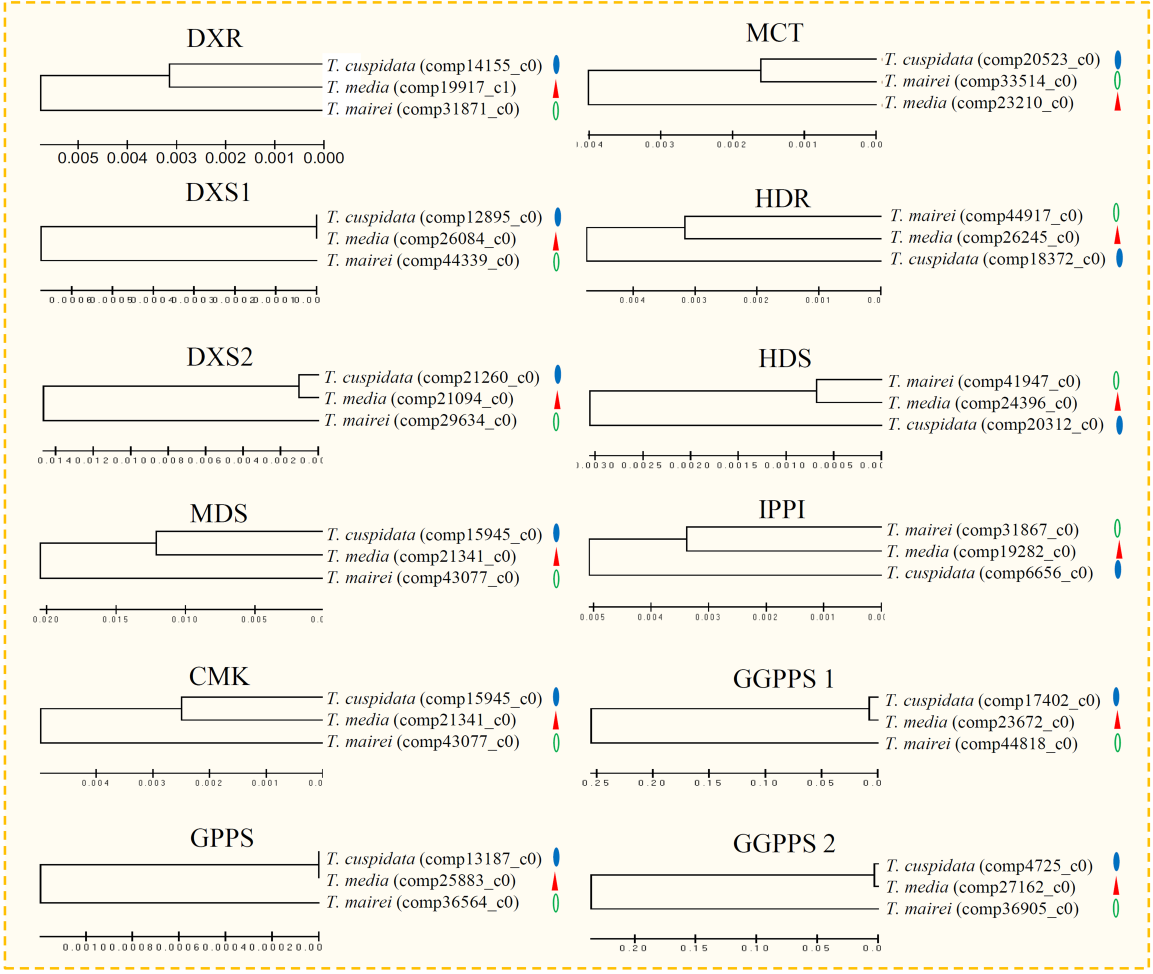


Figure S2 **Phylogenetic analysis of the proteins associated with the MEP pathway.** Phylogenetic trees of each enzyme associated with the MEP pathway were constructed using full length sequences. Enzymes abbreviations are: DXS: 1-Deoxy-D-xylulose 5-phosphate synthase; DXR: 1-Deoxy-D-xylulose 5-phosphate reductoisomerase; MCT: 2-C-methyl-D-erythritol 4-phosphate cytidylyltransferase; CMK: 4-(Cytidine 5-diphospho)-2-C-methyl-D-erythritol kinase; MDS: 2-C-methyl-D-erythritol 2,4-cyclodiphosphate synthase; HDS: 4-Hydroxy-3-methylbut-2-enyl-diphosphate synthase; HDR: 4-Hydroxy-3-methylbut-2-enyl diphosphate reductase; IPPI: isopentenyl diphosphate isomerase; GGPPS: geranylgeranyl diphosphate synthase.
