# Supplementary material for: Transcriptome analyses provide insights into the expression pattern and sequence similarity of several taxol biosynthesis-related genes in three Taxus species
Source: BMC Plant Biol. 2019 Jan 21;19:33. doi: 10.1186/s12870-019-1645-x (PMC6341696; doi:10.1186/s12870-019-1645-x)
Supplement: Supplementary file 9 — Figure S3. A heatmap of differential expressed TF genes in the three comparisons. (DOCX 283 kb) [file 12870_2019_1645_MOESM9_ESM.docx]

Figure S3 A heatmap of differential expressed TF genes in the three comparisons.
